# Shedding light on the problem: Influence of the radiator power, source-sample distance, and exposure time on the performance of UV-C lamps in laboratory and real-world conditions

**DOI:** 10.1371/journal.pone.0302258

**Published:** 2024-04-16

**Authors:** Katarzyna Arkusz, Kamila Pasik, Aleksandra Jędrzejewska, Tomasz Klekiel, Waldemar Woźniak, Marta Nycz, Roman Stryjski

**Affiliations:** 1 Department of Biomedical Engineering, Faculty of Mechanical Engineering, University of Zielona Gora, Zielona Gora, Poland; 2 Department of Production and Transport Engineering, Faculty of Mechanical Engineering, University of Zielona Gora, Zielona Gora, Poland; SKUMS: Shahrekord University of Medical Science, ISLAMIC REPUBLIC OF IRAN

## Abstract

Effective surface disinfection is crucial for preventing the spread of pathogens in hospitals. Standard UltraViolet-C (UV-C) lamps have been widely used for this purpose, but their disinfection efficiency under real-world conditions is not well understood. To fill this gap, the influence of the power of the ultraviolet radiator, source-sample distance, and exposure time on the performance of UV-C lamps against *Escherichia coli* and *Staphylococcus epidermidis* were experimentally determined in the laboratory and hospital. The obtained results showed that the UV irradiance and, thus, the UV-C disinfection efficiency decreased significantly at distances greater than 100 cm from the UV-C lamp. Moreover, increasing the total power of the radiators does not improve the performance of UV-C lamps under real conditions. The UV-C disinfection efficiency greater than 90% was achieved only under laboratory conditions at a close distance from the UV-C lamp, i.e., 10 cm. These findings provide novel insights into the limitations of UV-C lamps in real-world conditions and highlight the need for more effective disinfection strategies in hospitals.

## Introduction

The emergence of COVID-19 resulted in large-scale scientific, economic, and public efforts to contain viral transmission. Moreover, approximately 15% of all hospitalized patients suffer from nosocomial infections [1]. Viral and bacterial infection risk could be reduced through many control techniques, such as chemical disinfectants, heat sterilization, and UltraViolet (UV) radiation. UV radiation is divided into three categories: UV-C (100–280 nm), UV-B (280–315 nm), and UV-A (315–400 nm); however, the UV-C spectrum is credited with the biocidal effect. Hence, UV-C irradiation disinfection has been a fast-growing technology. However, the limited understanding of critical aspects of UV-C disinfection, not only among the general public but also among some disinfection manufacturers, has led to the misuse of this promising technology. Over the past few years, only a few reports refer to validated kinetic data on UV dose requirements for incremental log deletion but rather present time-based data [3–10, 13–14].

UV-C radiation’s effectiveness in surface disinfection decreases as the distance between the light source and the contaminated surface increases. The surface structure is another factor affecting the UV-C disinfection efficiency, with a hard, smooth surface having the most significant impact [2, 3]. High disinfection efficiency of UV-C lamps was demonstrated in 5–60 seconds at a very close distance from the lamp, i.e., 4 cm [4], 3–8 cm [5], 10 cm [6], 25 cm [7], 30 cm [8], 34 cm [9]. However, these conditions in no way reflect actual hospital conditions.

So far, few literature reports indicate the biocidal efficacy of UV-C radiation in the healthcare environment. The performance of UV-C lamps in a hospital room determined by Rutala *et al*. showed that 99.8% of *Candida*, ~95% of *Staphylococcus aureus* and *Enterococcus* were eliminated after 50-minutes exposure to UV-C radiation dose ranged from 4720 J/m^2^ to 6610 J/m^2^ [10]. Similar effectiveness of UV-C disinfection was also confirmed by Casini *et al*., who reported a maximum total reduction of bacteria in the patient room was 82%, 92% in intensive care units, and 92% in the operating room with low turnover [11]. However, Hakim *et al*. performed a more detailed analysis and indicated the UV-C disinfection efficiency in hospital rooms in the wide range from 10 to 85%, depending on the sample location [12]. Other research [13, 14] also indicated a bacterial reduction greater than 90% in a patient room, emphasizing the inability to use this method in crowded wards or large open spaces, in shadowed areas (e.g., bedrail, bedside, telephone) and in the regions which were covered with dirt or debris. Based on these studies, the effectiveness of UV-C disinfection under real-world conditions can not be determined, which is crucial for patient safety.

Therefore, the aim of this study was to perform comprehensive tests determining the UV-C disinfection efficiency under real-world conditions: in the laboratory and healthcare environment. The number of variables tested is also innovative, such as the impact of the nominal power of the ultraviolet radiator, the distance from the UV-C lamp, and the exposure time to UV-C radiation on the disinfection efficiency. The UV-C disinfection efficiency was determined based on microbiological tests against Gram (+) *Staphylococcus epidermidis* and Gram (-) *Escherichia coli* bacteria with continuous monitoring of the dose of UV-C radiation. To the best of our knowledge, this is the first such extensive study on the effectiveness of UV-C disinfection. The conducted research will allow a quantitative comparison of the performance of UV-C lamps under laboratory and real conditions and will also allow the determination of new disinfection procedures.

## Materials and methods

**Fig 1:**
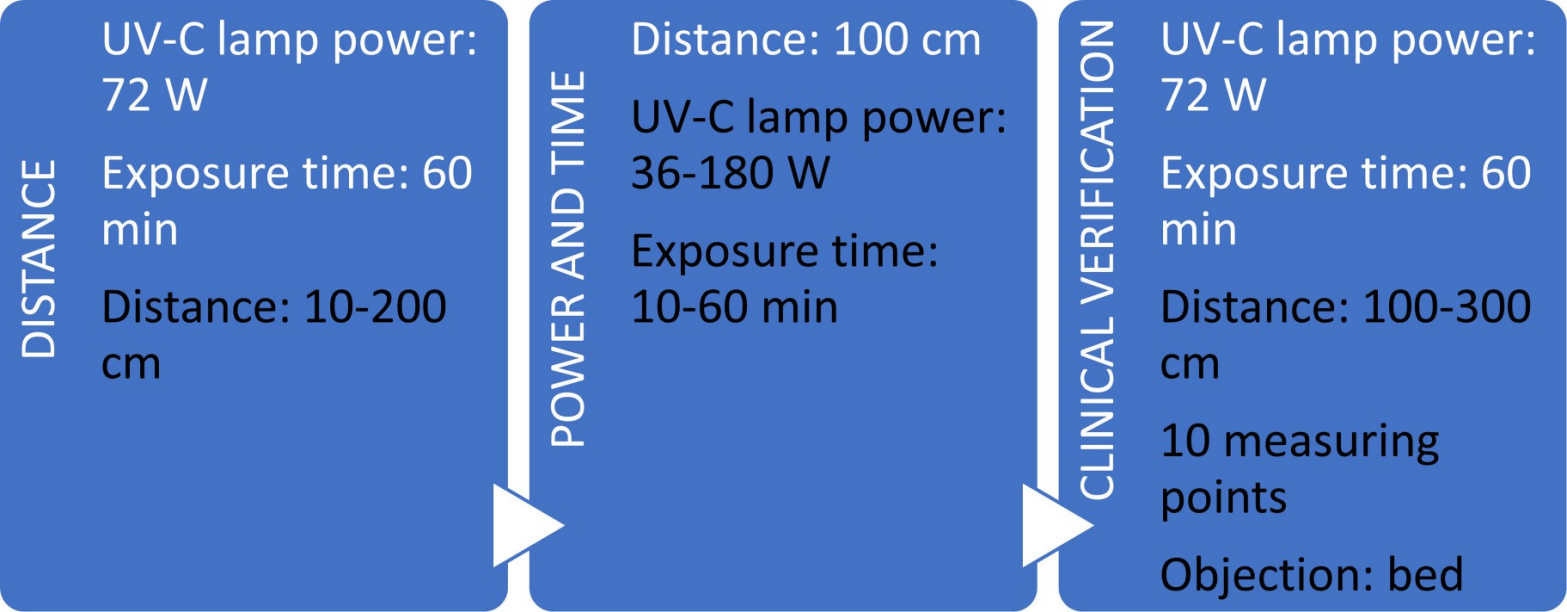
Schema of research determining the disinfection efficiency using UV-C radiation, considering the distance from the radiation source, the power of UV-C lamps, and the duration of UV-C exposure.

To investigate the effects of the total power of the UV-C lamp, the distance between the UV-C source and surface, and different UV-C exposure times, the UV-C disinfection efficiency was calculated based on microbiological tests performed according to the research program shown in Fig 1. The scheme demonstrates three main phases:

**Stage 1**: determining the effect of the distance from the UV-C source to the analyzed sample on the disinfection efficiency. The research was carried out under laboratory conditions using a UV-C device with the power of 72 W radiators, irradiating plates with seeded bacteria for 60 minutes. The samples were placed at 10–200 cm distances, with simultaneous radiation dose measurements.

**Stage 2:** determining the influence of radiation dose (regulated by changing the power of the radiators) and the duration of exposure to UV-C radiation on disinfection efficiency. The microbiological samples were placed 100 cm away from the UV-C device, which imitates the actual place of installation of the UV-C lamps in the hospital and the shortest distance from the hospital equipment. The variable parameters at this research stage were the power of the radiators in the range from 36 W to 180 W and the irradiation time of 10–60 minutes.

**Stage 3**: determining the disinfection efficiency using the UV-C device in a laboratory room mapping a hospital room on a 1:1 scale, in which the samples were distributed in 10 measurement locations considering the obstacle of the hospital bed. The exposure time with a 72 W UV-C lamp was 60 minutes. The conditions in the room were a temperature of 20.5 ± 0.5°C and relative humidity (RH) of 46.5 ± 1%.

**Fig 2:**
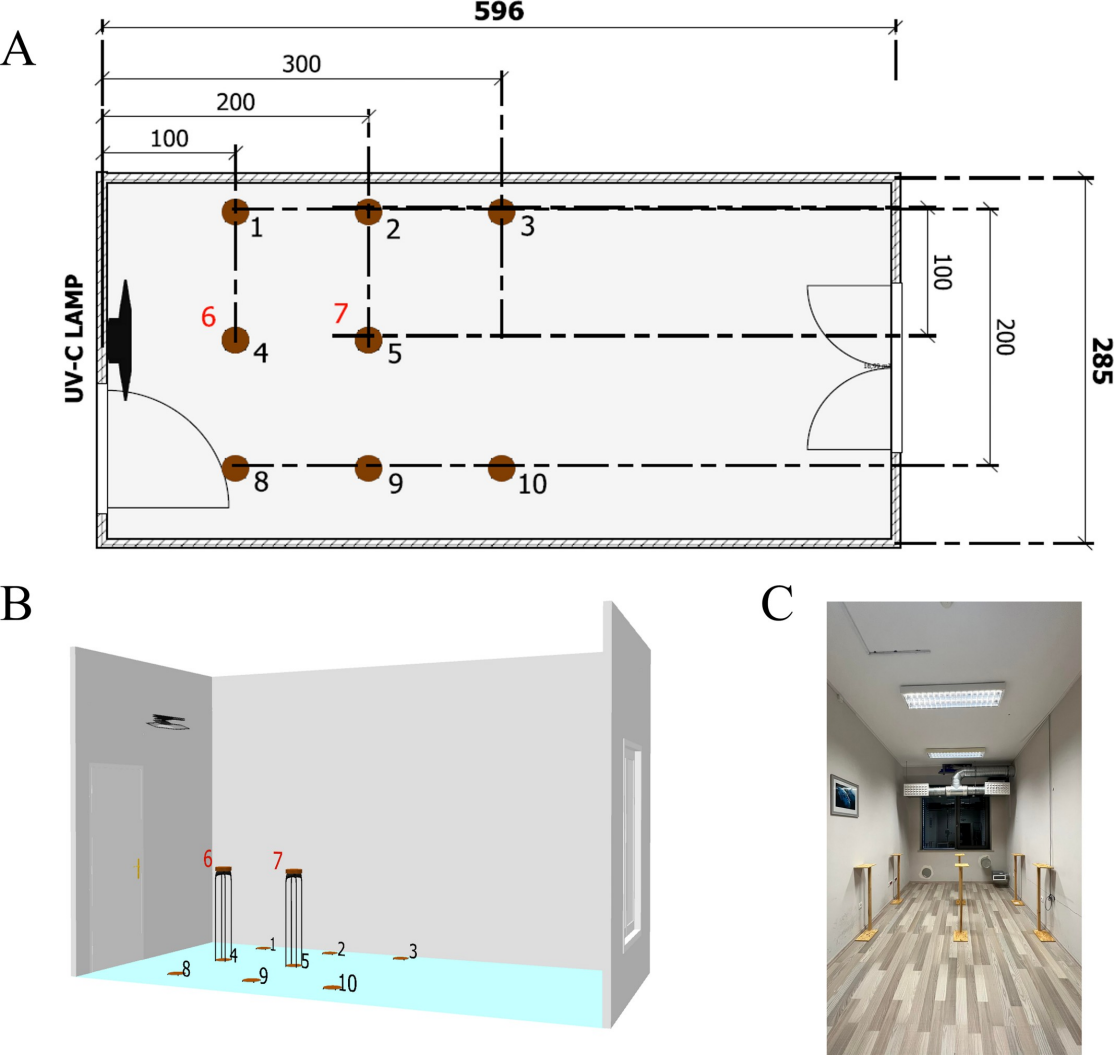
Schema of the representative patient’s room in (A) plan view, (B) 3D view, and (C) real view showed the positioning of the UV-C lamp and area of sampling. The UV-C device was placed under the entrance to the room at a height of 250 cm. The samples were placed on the floor at a distance of 100 cm (Fig 2A, points 1, 4, 8), 200 cm (Fig 2A, points 2, 5, 9), and 300 cm (Fig 2A, points 3, 10). Two samples, no. 6 and 7, were placed at the height of 100 cm at a distance of 100 cm and 200 cm from the UV-C lamp, respectively.

All measurements were performed in the laboratory and clinical room, imitating hospital conditions (Fig 2). The research was carried out using the UV-C device described in 2.2. section in the research room in the Center for Sustainable Building and Energy in the Science and Technology Park of the University of Zielona Gora in Nowy Kisielin. The view of the room with dimensions (length x width x height) of 596 cm x 285 cm x 320 cm, together with the locations of the distribution of test samples, is shown in Fig 2.

### Strains and microbial conditions

The bacterial strains used to assess the disinfection efficiency of UV-C radiation were *Escherichia coli (E*. *coli)* and *Staphylococcus epidermidis (S*. *epidermidis)*, as representative of Gram-negative and Gram-positive, respectively. They are typical bacteria commonly found in dust and hospital environments [1] and have been used in previous UV disinfection experiments [5, 15]. *E*. *coli* ATCC 11229 and *S*. *epidermidis* ATCC 35984 strains were purchased as a KWIK-STIK™ from the Pol-Aura, Poland.

The antimicrobial activity was conducted following the broth microdilution method, following the National Committee on Clinical Laboratory Standards (NCCLS) guidelines. In detail, *E*. *coli* and *S*. *epidermidis* strains were initially incubated at 37°C for 24 h on CASO medium–Tryptic Soya Agar (no. PA-24-CM0131B, Pol-Aura, Poland) and on Chapman medium–Mannitol Salt medium (no. PA-24-CM0085B, Pol-Aura, Poland), respectively. Next, the inoculum was prepared. *E*. *coli* strains were suspended in the CASO medium and *S*. *epidermidis* in the Chapman medium to the optical density OD_600_ = 0.025±0.005. A total of 1 mL of bacterial inoculum was added to a sterile 90-mm petri dish and exposed to UV light. After exposure to the UV-C light, the Petri dish was sealed and placed in an incubation chamber for 24 hours at 37°C. Petri dishes with 1 mL of bacterial inoculum, kept in the same condition with the UV-C lamp turned off and after 24-hours incubation at 37°C, were used as blank reference samples. All experiments were performed independently fivefold, and the results were expressed as mean ± standard deviation.

### Ultraviolet C device

The UV-C device used in all presented tests was an open-type lamp equipped with 120 cm 36 W 8-inch diameter tubular (T8) standard low-pressure mercury vapor discharge lamps (Philips Lighting, Netherlands), powered from the wall outlet (230 VAC 50 Hz) via electronic ballast. The device consists of a lamp array and a mirrored surface from the back of the array to guide emitted radiation toward the front of the device. The peak emission of the UV-C device was measured using a wide-band semiconductor spectroradiometer (Gigahertz-Optik GmbH., Germany) and was confirmed to be at 253.7 nm.

### Data reduction

To compare the performance of the UV-C lamp under different conditions, the UV-C disinfection efficiency of the direct UV-C lamp was defined as follows [14, 16]:

$$UV-C\;disinfection\;efficiency=\frac{{CFU}_{i,lamp=on}}{{CFU}_{i,lamp=off}}$$ (1)

where *CFU_i,lamp = on_* and *CFU_i,lamp = off_* represent the colony-forming units of bacteria *i* under UV-C lamp-on and lamp-off conditions, respectively.

## Results

The disinfection efficiency of the commercially available UV-C device was tested against *E*. *coli* (ATCC 11229) and *S*. *epidermidis* (ATCC 35984), taking into account the UV-C source-sample distance, time of irradiation, and power of radiators.

**Fig 3:**
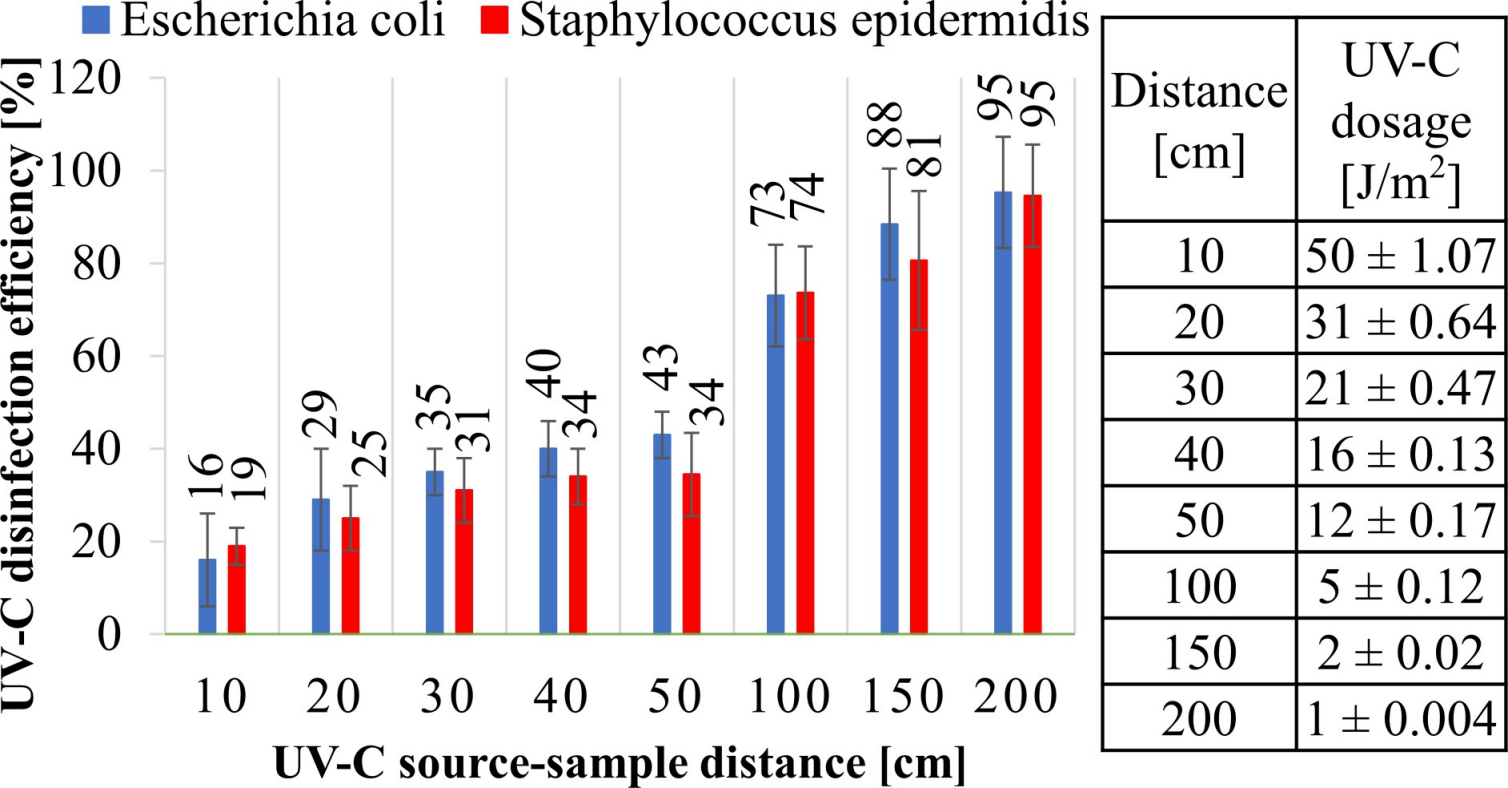
The effectiveness of the UV-C device (72-watt) in reducing *Escherichia coli* and *Staphylococcus epidermidis* after treatment for 60 minutes at varying distances between the sample and the UV-C source (0–200 cm).

Stage no. 1: Increasing the UV-C distance from the lamp reduces the radiation dose and significantly reduces the UV-C disinfection efficiency (Fig 3). For example, 10 cm from the UV-C lamp, the UV-C dose was 50 ± 1.07 J/m^2^, and the disinfection efficiency was 84% (against *E*. *coli)* and 81% (against *S*. *epidermidis)*. Increasing the distance to 20 cm reduced the UV-C radiation’s emitted dose to 31 ± 0.64% and the UV-C disinfection efficiency to 71% and 75%, respectively. For more than 100 cm, the UV-C disinfection efficiency is minimal: at 100 cm, it is 25%, and for a distance of 150 cm, it is higher than 10%, while for a distance of 200 cm, it is insignificant at about 5%.

**Fig 4:**
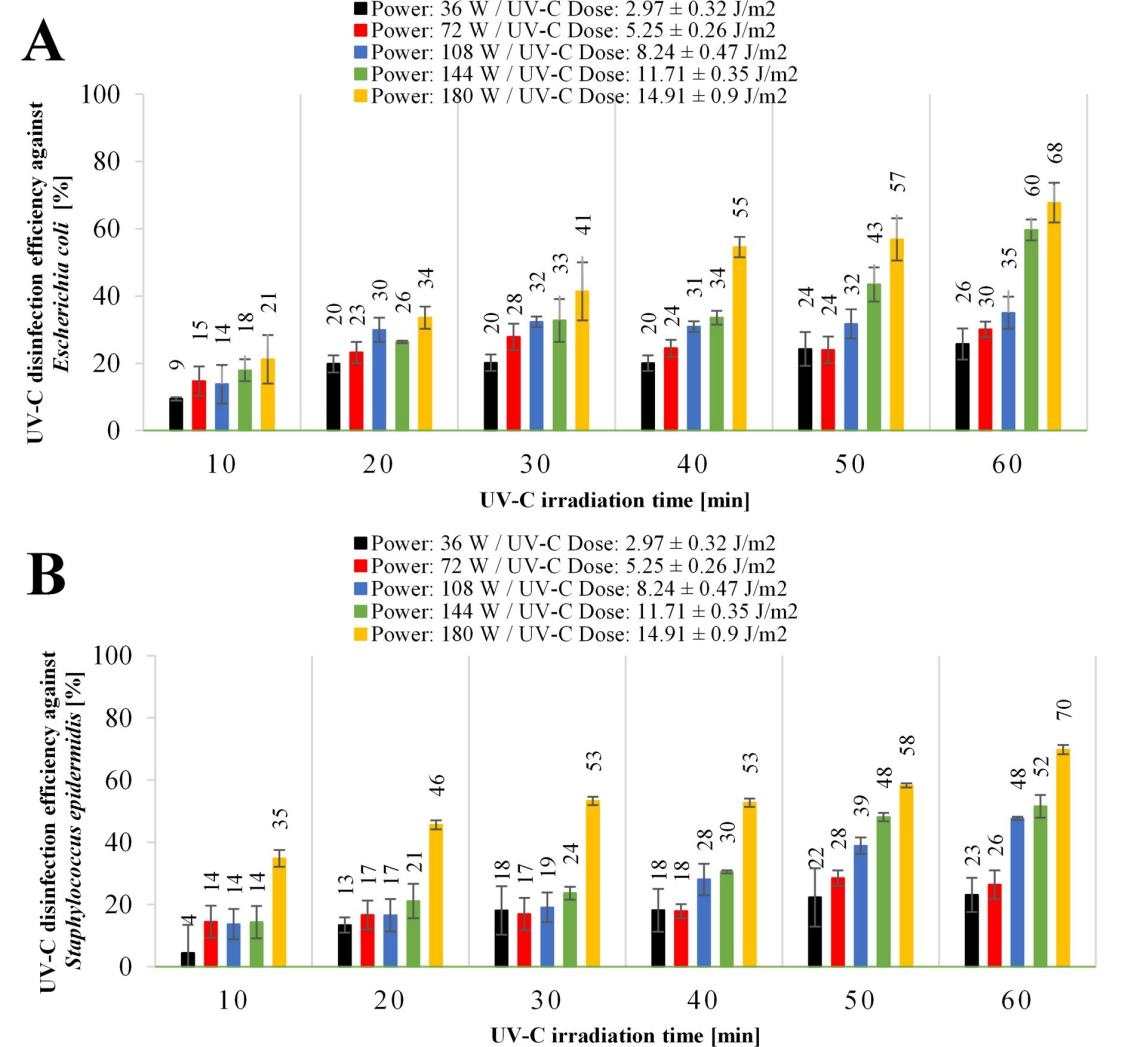
UV-C disinfection efficiency measured against (A) *Escherichia coli* and (B) *Staphylococcus epidermidis* exposed to different nominal power of UV-C radiator (36–180 W) at 100 cm distance and different irradiation times (10–60 min).

Stage no. 2: the inactivation of *E*. *coli* and *S*. *epidermidis* was investigated by varying power of the UV-C device (36–180 W) placed 100 cm from the sample and different irradiation times (10–60 min) (Fig 4). UV-C irradiation for 0–60 min resulted in UV-C disinfection efficiency against *E*. *coli* proportional to the power of the UV-C radiators (Fig 4A). The UV-C disinfection efficiency against *E*. *coli* after 60-minute exposition to a 36 W UV-C radiator was 26%, to 72 W UV-C radiator was 30%, to a 108 W UV-C radiator was 35%, to a 144 W UV-C radiator was 60%, and to a 180 W UV-C radiator was 68%. A 36-watt UV-C radiator enables just a 23% disinfection efficiency in reducing *S*. *epidermidis* after 60 minutes of irradiation (Fig 2B). When the UV-C radiator’s power is increased further to 72 W, the disinfection efficiency increases up to 26%, up to 48% for 108 W UV-C radiators, up to 52% for 144 W UV-C radiators, and up to 70% for 180 W UV-C radiators while maintaining an exposure time of 60 minutes.

Stage no. 3: The disinfection efficiency of the UV-C device in real conditions calculated based on the reduction of *E*. *coli* and *S*. *epidermidis* in 10 selected points is shown in Table 1. At each point, colony forming unit (CFU) reduction of *E*. *coli* and *S*. *epidermidis* was observed after exposure to UV-C radiation emitted by a UV-C lamp with a power of 72 W for 60 minutes. The highest UV-C disinfection efficiency was recorded for sample no. 6, i.e., the closest point to the UV-C device, with an 80% reduction (Log_10_ reduction = 0.699) in *E*. *coli* and a 71% reduction (Log_10_ reduction = 0.5376) in *S*. *epidermidis*. On the other hand, the lowest biocidal efficiency was demonstrated at points most distant from the source of UV-C radiation, i.e., in point no. 10, UV-C disinfection efficiency was 33% against *E*. *coli* (Log_10_ reduction = 0.17393) and 19% against *S*. *epidermidis* (Log_10_ reduction = 0.09151). Similarly, in point no. 3, UV-C disinfection efficiency was 36% (Log_10_ reduction = 0.19382) and 41% (Log_10_ reduction = 0.22915) against *E*. *coli* and *S*. *epidermidis*, respectively. A low UV-C disinfection efficiency against *E*. *coli* and *S*. *epidermidis* was also recorded for points 4 and 5, i.e., places closest to the radiation source but distributed on the floor.

**Table 1:** UV-C disinfection efficiency measured in different places in the patient room (Fig 2) after 60 minutes of irradiation.

Number of sampling (length;width;height) [m]	UV-C dose per second [J/m^2^]	UV-C disinfection efficiency against *E*.* coli* [%]	UV-C disinfection efficiency against *S*.* epidermidis* [%]
1 (0.25; 1; 0)	0.579 ± 0.046	73 ± 6	62 ± 5
2 (0.25; 2; 0)	0.374 ± 0.029	72 ± 2	71 ± 9
3 (0.25; 3; 0)	0.209 ± 0.017	36 ± 1	41 ± 4
4 (1.25; 1; 0)	0.642 ± 0.051	61 ± 6	28 ± 1
5 (1.25; 2; 0)	0.404 ± 0.032	63 ± 5	28 ± 3
6 (1.25; 1; 1)	0.976 ± 0.078	80 ± 5	71 ± 6
7 (1.25; 2; 1)	0.469 ± 0.037	77 ± 4	47 ± 3
8 (2.25; 1; 0)	0.446 ± 0.036	71 ± 1	47 ± 4
9 (2.25; 2; 0)	0.305 ± 0.024	71 ± 6	63 ± 7
10 (2.25; 3; 0)	0.210 ± 0.017	33 ± 4	19 ± 1

**Fig 5:**
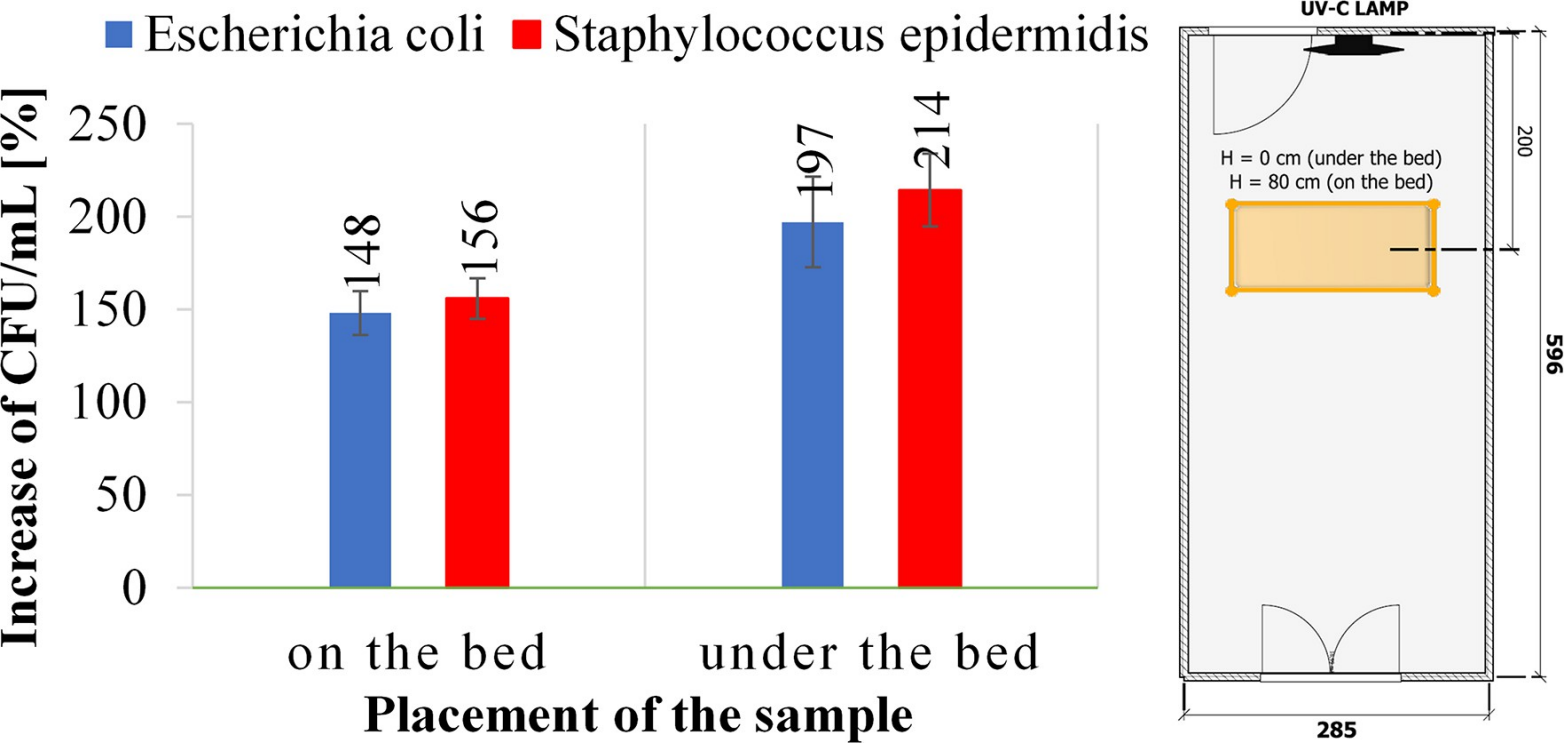
The influence of furniture on the disinfection efficiency of a UV-C device against *Escherichia coli* and *Staphylococcus epidermidis* after 60 minutes of UV-C irradiation.

The penetration of UV-C radiation through the hospital bed was analyzed using a 72-watt UV-C device during the exposition of the sample for 60 minutes (Fig 5). The obstacle in the form of a hospital bed caused a complete lack of effectiveness of UV-C disinfection against both types of bacteria. The total UV-C disinfection efficiency against *E*. *coli* and *S*. *epidermidis* increased by 48% and 56%, respectively, for the samples placed on the bed and by 97% and 114% for the samples placed under the bed compared to a reference sample.

## Discussion

Microbiological studies to determine the effectiveness of UV-C disinfection were carried out using *Escherichia coli* and *Staphylococcus epidermidis*, which are the leading causes of nosocomial infections in various parts of the world [17, 18]. The influence of intermediate parameters of the surface disinfection process with the use of UV-C radiation, i.e., the distance of the disinfected surface from the UV-C radiation source (Fig 3), the wattage of the UV-C device and the irradiation time (Fig 4), was analyzed. Furthermore, the key parameter for reducing microorganisms, the UV-C radiation dosage, was considered. As the distance from the 72-watt UV-C device increases, the emitted UV-C radiation dose decreases, and thus the effectiveness of bacterial inactivation decreases. The highest UV-C disinfection efficiency was observed at 10 cm from the 72-watt UV-C device, respectively 84 ± 10% for *E*. *coli* and 81 ± 4% for *S*. *epidermidis*. The greater reduction in *E*. *coli* results from the difference in the cell wall structure, which for Gram-negative bacteria is much thinner and has a thickness of 2–3 nm, compared to Gram-positive bacteria, whose peptidoglycan wall is 10–80 nm thick [19]. Therefore, UV-C radiation penetrates the bacterial structure, changing the structure of DNA. As the distance from the UV-C radiation source increased (Fig 3), an exponential decrease in radiation dose and decreased efficiency of *E*. *coli* and *S*. *epidermidis* inactivation was observed. At a distance of 200 cm, the effect of UV-C radiation on surface disinfection is almost unnoticeable.

According to data from the literature, the irradiation (surface dose density) of UV-C radiation necessary for 90% (Log reduction—1) of the destruction of various organisms (D_90_) is 25 J/m^2^ for *E*. *coli* and 21 J/m^2^ for *S*. *epidermidis*. In particular, biocidal efficiency at the level of 99.99% of E. coli (strain K12) is possible at 30–125 J/m^2^ [20], and S. epidermidis is possible at 170 J/m^2^ [21]. The measured UV-C radiation dose generated by the 72-watt UV-C device falling perpendicularly to the sample with bacteria reaches 50 ± 1.07 J/m^2^ at 10 cm. Theoretically, this dose is required to inactivate *E*. *coli* and *S*. *epidermidis*. However, the efficiency of the UV-C disinfection process is significantly lower than expected.

Literature data on assessing the UV-C disinfection efficacy indicate significant discrepancies in the UV-C radiation doses necessary to reduce microorganisms effectively [15, 20]. Cheng *et al*., for the tested UV-C device, indicated a very promising surface disinfection efficiency using a 1 cm distance from the lamp and a UV-C irradiation dosage of 119 J/m^2^ treatment on nutrient-rich surfaces and a 5-log reduction (99.999%) using UV-C dosage of 238 J/m^2^ on surfaces in both wet and dry conditions [15]. In the studies of Santos *et al*., 99.9% reduction efficiency of *E*. *coli* and *S*. *epidermidis* was obtained using a distance from the lamp of 1 cm and an exposure time of 20 s, which allowed to obtain a dose of 254 nm UV-C light dose was 9120 J/m^2^ in both conditions (in vitro and hospital) [22]. These discrepancies confirm that the assessment of UV-C radiation’s effectiveness solely on the emitted radiation dose is insufficient. In addition, these studies demonstrate that the high performance of the UV-C device, providing a reduction of 99.9%, is only observed at very close distances from the lamp, i.e., less than 10 cm [4–6, 23]. Additionally, increasing the exposure time using a low dose of radiation is not the only parameter influencing the effectiveness of surface disinfection using UV-C radiation, especially in real conditions [24, 25]. Increasing the exposure time with a small dose of radiation does not inhibit the multiplication process of bacteria, and they can replicate even in the presence of induced mutations [23].

Therefore, to confirm the above conclusion, the influence of UV-C lamps’ power and exposure time on the disinfection efficiency was tested in laboratory conditions (Fig 4). A distance of 100 cm of samples from the UV-C irradiation source was used. The UV-C devices with power in the range of 36–180 W were tested, with an exposition time of 10–60 min, and the results in the reduction of *E*. *coli* and *S*. *epidermidis* were shown in Fig 4. The UV-C radiation dosage emitted by the 36-watt UV-C lamp during 10 min was 1782 J/m^2^, and the highest dose of 53676 J/m^2^ was emitted by the 180-watt UV-C lamp and the exposure time of 60 minutes. Under these extreme conditions, UV-C disinfection efficiency against *E*. *coli* was 9–68%, and against *S*. *epidermidis* was 4–70%. The results of the presented research suggest that the 72-watt UV-C device used in practice, installed in health care units, is insufficient for effective surface disinfection, taking into account the distance of 100 cm [26]. It should be emphasized that the assumed distance of 100 cm from the UV-C device is the minimum distance from the UV-C device in real conditions (e.g., hospital rooms).

Furthermore, the effectiveness of the UV-C radiation method in surface disinfection was verified in a 1:1 scale laboratory room (Fig 2) corresponding to a hospital room, and the results are shown as *E*. *coli* and *S*. *epidermidis* reduction in Table 1. The results showed the highest efficiency of surface disinfection for samples located in the line of UV-C radiation. The disinfection efficiency against *E*. *coli* and *S*. *epidermidis* at a distance of 100 cm from the UV-C device was 29–80% (samples no. 1, 4, 6, 8), whereas the lowest inactivation was measured on the floor. Disinfection with UV-C radiation showed a bacterial inactivation of 28–77% at a distance of 200 cm from the UV-C device; again, the lowest reduction was observed in the sample on the floor. 19–41% of the bacteria were inactivated by UV-C radiation at a distance of 300 cm from the UV-C device. The dose of emitted UV-C radiation per second corresponded to the radiation efficiency (Table 1).

The UV-C radiation received in different areas varied between 0.209 ± 0.046 J/m^2^ and 0.976 ± 0.078 J/m^2^ (median 0.425 J/m^2^). During the 60-minutes exposition, the UV-C dosage was between 752 J/m^2^ and 3513 J/m^2^. Surfaces at shorter distances and in the direct line of sight of the UV-C device showed statistically significantly higher UV-C doses than surfaces in the shadow of the equipment. Lindblad *et al*. also investigated UV-C dose received in a hospital room, where the measured UV-C dose emitted by a mobile automated UVC light-emitting decontamination with the sporicidal setting of 22000 mWs/cm^2^ was in the range of 160–10680 J/m^2^ at the distance from the light source in the range of 97–502 cm [23]. However, the authors did not perform microbiological tests, and the effectiveness of UV-C disinfection was demonstrated based on literature doses of UV-C radiation necessary to inactivate microorganisms. Presented in this paper and other [12, 27] research has shown that the biocidal efficiency of UV-C radiation is much lower under real conditions. The explanation of the above-described results of our research and those quoted from the literature show lower effectiveness of UV-C disinfection in practice, which may be explained by the defense mechanisms helping bacteria survive. Bacterial repair mechanisms such as photoreactivation, excision repair, mismatch repair (MMR), double-strand gap repair (DSB) and other mechanisms such as dimer bypass, SOS response (save our soul), point activation checkpoints, and programmed cell death (PCD) or apoptosis, which effectively remove changes in DNA, ensuring the integrity of the genome, help them continue to reproduce after exposure or during exposure to UV-C radiation [28]. Moreover, it was confirmed that the form of *E*. *coli* bacteria, under the influence of UV-C radiation, is made up of a strikingly densely packed, dome-like outer shell at the liquid-air interface of the droplet, while the inner core of the liquid droplet contains life cells [15].

The last stage of the research was to verify the effectiveness of surface disinfection using UV-C radiation, considering the furniture in the room. The obtained results (Fig 5) clearly showed that the room’s furnishing reduces the effectiveness of the UV-C surface disinfection process. Furthermore, UV-C radiation does not reach the area under the bed, which poses a severe risk for the multiplication and transmission of microorganisms on footwear, increasing the epidemiological risk. In particular, a reduction in microbial reduction was also observed on the bed surface. Due to the above considerations, the dose of UV-C radiation necessary to inactivate pathogens in fully equipped hospital rooms should be significantly increased. Moreover, the classic placement of the UV-C device on the walls does not disinfect the floor and shadowed places. Numerous studies also demonstrated that UV-C was not effective in shadowed areas of rooms, necessitating further disinfection [13, 16, 22, 28–33]. UV-C light had been shown to inactivate infectious microorganisms in other areas (i.e., stethoscope, ambulance, respirator). However, the time required to disinfect was at least 15 hours in the areas not in line with UV-C light (18-watt) [34].

It is worth emphasizing that all relevant studies were included in this discussion. However, the difficulty/limitation of a quantitative comparison results from the significant heterogeneity of the methodology due to the lack of standards and probably different sterilization protocols in various healthcare facilities.

## Conclusions

The effectiveness of direct-type UV-C devices for surface disinfection against *Escherichia coli* and *Staphylococcus epidermidis* was investigated in this study. The influence of distance from the UV-C radiation source, power of the radiators, exposure time, and shading on the UV-C radiation dose and disinfection efficiency using the UV-C method was determined. It was demonstrated that disinfection efficiency greater than 90% could be achieved under real-world conditions by placing the UV-C source less than 10 cm from the surface to be disinfected, using a minimum 72 W radiator, and irradiating for 60 minutes. As the distance from the UV-C source increased, a lower dose of UV-C radiation and lower disinfection effectiveness were observed. For a reference distance of 100 cm, a radiator power greater than 180 W was necessary to achieve 90% (Log reduction—1) disinfection efficiency for a 60-minute exposition. Additionally, it was found that UV-C disinfection efficiency was highest along the radiation line and did not pass through equipment. It is worth noting that the main limitation of this type of research is the difficulty of referring their results to unpredictable conditions that occur in a live setting (high-traffic, high-polluted, high-touch places like health care facilities), and therefore of controlling confounding variables that may lead to an overestimation of the effectiveness of UV-C irradiation. Another difficulty is referring the obtained results to the results of other research teams due to the significant diversity of methodology, which may result from differences in the protocols used in the case of UV-C disinfection. The results indicate the low efficiency of commercially available UV-C direct-type wall lamps and suggest using mobile and self-steering solutions that reduce the distance between the radiation source and the surface to be disinfected. Future research will focus on examining and analyzing the effectiveness of such an innovative solution that we want to propose.
